# A case of extraocular sebaceous carcinoma with unusual dermoscopic features: Vincent Van Gogh's Starry Night landscape

**DOI:** 10.1111/srt.70049

**Published:** 2024-10-04

**Authors:** Erhan Ayhan, Sulin Temmo

**Affiliations:** ^1^ Department of Dermatology Dicle University Faculty of Medicine Diyarbakır Turkey

Dear Editor,

A 49‐year‐old man with a 3‐year history of itchy mass on the left upper forehead (Figure 1A). Dermoscopic examination revealed irregular pigment network and concentric pigment rings on a yellowish background. In addition, yellowish‐white structure less areas, telangiectasias, brown follicular openings and yellowish‐white rings surrounding these openings were observed (Figure 1B). However, since pigmented structures could not be clearly evaluated under intense light, they were clarified using a mobile photo editor application (MPEA) (Figure 1C). This image was partially resembled Vincent Van Gogh's Starry Night landscape. Immunohistochemically, the tumor stained pan‐cytokeratin positive, CD117 positive, BER‐EP4 focal positive, epithelial membrane antigen luminal positive. DOG1 stained weakly diffuse positive. When all findings were evaluated, the patient was diagnosed as sebaceous carcinoma (SC).

**FIGURE 1:**
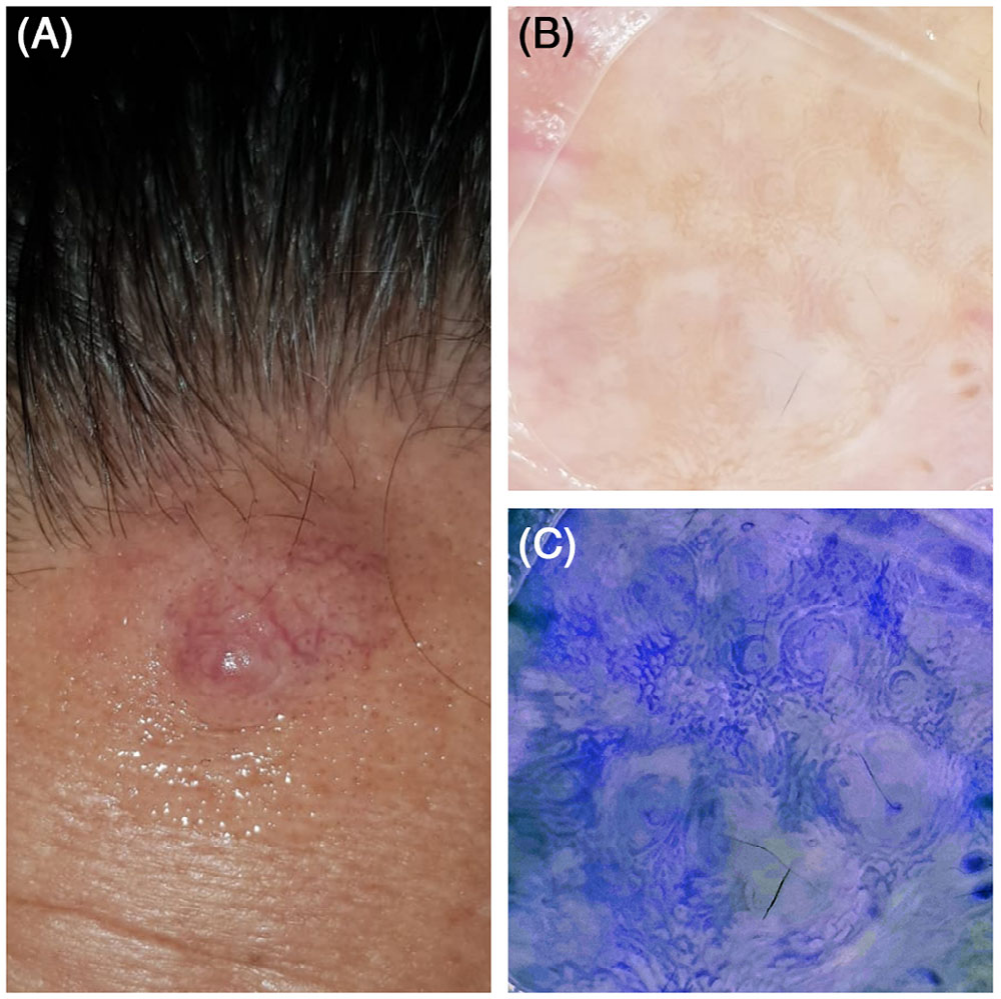
(A) In the upper left region of the frontal region, a sharply circumscribed, round‐shaped, smooth‐surfaced, dome‐shaped nodular lesion measuring approximately 1 × 1.5 cm with telangiectasia is seen. (B) Pigmented structures that cannot be clearly distinguished. (C) It is seen that the pigment circles become clearly visible with the photo editor application.

In a dermoscopic study of 15 patients with SCs, the most frequently observed structures included polymorphous vessels, whitish‐pink areas, yellowish structures and yellowish structureless areas. Pigment network has been detected in non‐melanocytic lesions such as dermatofibroma, cutaneous mastocytosis, syringoma, pigmented purpuric dermatosis, pigmented seborrheic keratosis.^1^ There are previous case‐level publications on clarification of dermoscopic images with a MPEA.^2^, ^3^ In our case, the pigmented dermoscopic structures were swirl‐shaped and a MPEA was used to visualize these structures more clearly.

In conclusion, swirl‐like pigment rings can be seen in SC and the use of a MPEA can be useful for better definition of dermoscopic structures.

## CONFLICT OF INTEREST STATEMENT

The authors declare no conflicts of interest.

## Data Availability

The data that support the findings of this study are available from the corresponding author upon reasonable request.
